# Vaccines and Therapies in Development for SARS-CoV-2 Infections

**DOI:** 10.3390/jcm9061885

**Published:** 2020-06-16

**Authors:** David Wu, Raghuram Koganti, Upendra P. Lambe, Tejabhiram Yadavalli, Shyam S. Nandi, Deepak Shukla

**Affiliations:** 1 Department of Ophthalmology and Visual Sciences, University of Illinois at Chicago, Chicago, IL 60612, USA; dwu36@uic.edu (D.W.); rkogan3@uic.edu (R.K.); yteja@uic.edu (T.Y.); 2 National Institute of Virology, Indian Council of Medical Research, Mumbai 400012, India; upendralambe69@gmail.com (U.P.L.); nandibiotech@gmail.com (S.S.N.); 3 Department of Microbiology and Immunology, University of Illinois at Chicago, Chicago, IL 60612, USA

**Keywords:** SARS-CoV-2, therapies, vaccines, COVID-19, coronavirus

## Abstract

The current COVID-19 pandemic is caused by the novel coronavirus SARS-CoV-2. The virus causes severe respiratory symptoms which manifest disproportionately in the elderly. Currently, there are over 6.5 million cases and 380,000 deaths reported. Given the current severity of the outbreak, there is a great need for antiviral therapies and vaccines to treat and prevent COVID-19. In this review, we provide an overview of SARS-CoV-2 and discuss the emerging therapies and vaccines that show promise in combating COVID-19. We also highlight potential viral targets that could be exploited by researchers and drug manufacturers.

## 1. Introduction to SARS-CoV-2

The novel coronavirus SARS-CoV-2 originated in Wuhan, China and caused an outbreak in December 2019 [1]. By late January, cases were reported in the United Kingdom, Russia, Spain, Canada, and Sweden [2]. This led the World Health Organization (WHO) to declare a state of Global Public Health Emergency on 30 January 2020 [2]. As of 27 May 2020, the virus has spread to 210 countries, and confirmed cases have crossed 5.7 million [2]. Confirmed deaths have already exceeded 380,000, dwarfing the 774 deaths from SARS-CoV (SARS-classic) [3] and rivaling the 151,700–575,400 estimated deaths from the 2009 swine flu pandemic [4]. The United States currently leads the world in both confirmed cases (1.3 million) and deaths (79,773) [2].

SARS-CoV-2, previously known as 2019-nCoV, is a member of the family *Coronaviridae* and genus *Betacoronavirus* [5]. Betacoronaviruses are positive-sense, single-stranded RNA viruses that possess envelopes and relatively large genomes [5]. Both SARS-CoV and SARS-CoV-2 are betacoronaviruses [5]. SARS-CoV-2 is the seventh coronavirus to cause illness in humans [6].

SARS-CoV-2 is believed to have originated in bats and utilized pangolins as intermediate hosts before passing on to humans [1]. Transmission between humans occurs via contact from respiratory droplets [7]. Transmission also occurs through contact with contaminated surfaces; the virus has a median half-life of 5.6 h on stainless steel and 6.8 h on plastic [8]. Infections can result from contact with steel or plastic for up to 3 days after the surface was first contaminated [9]. Studies have estimated the R_0_ of SARS-CoV-2 to be between 2.28–5.7 which places it on a similar level as HIV (2–5) and smallpox (3.5–6) [10,11].

The incubation period of COVID-19, the disease caused by SARS-CoV-2 infections, is reported to be between 2–14 days [12]. Common symptoms include fever, cough, shortness of breath, headache, and myalgia [13]. Ocular manifestations of COVID-19 include epiphora, conjunctival congestion, and chemosis [14]. The Center for Disease Control (CDC) recommends to go to the emergency room immediately if any of the following symptoms develop: trouble breathing, persistent chest pain, confusion, and bluish lips [12]. Severe COVID-19 cases may require patients to be put on a ventilator until their breathing improves. The estimated mortality rate by the WHO is 3.4% as of May 2020 but varies greatly based on age, location, prior health status, and other risk factors. Because it is difficult to know the extent to which individuals are infected with the virus, the estimated mortality rate should be viewed cautiously.

## 2. Structure of SARS-CoV-2 Protein Targets and Comparison to SARS-Classic

**Figure 1:**
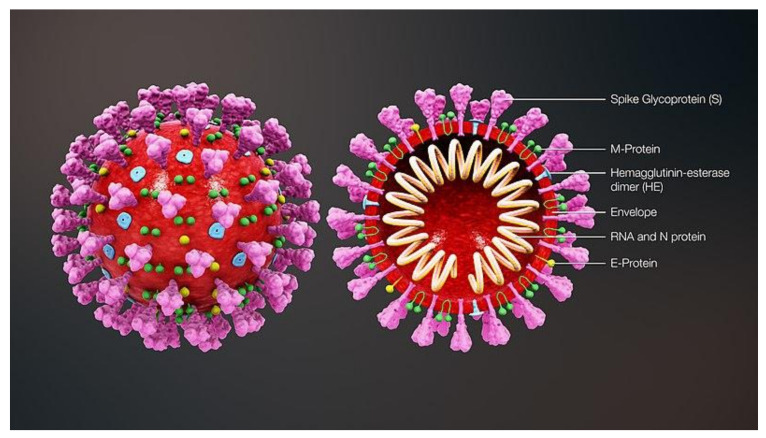
SARS-CoV-2 structure. The key structural proteins of the virus are listed. The spike (S) protein mediates attachment and fusion. The membrane (M) and envelope (E) proteins provide structure to the virion. The nucleocapsid (N) packages the viral single-stranded RNA genome. The hemagglutinin-esterase (HE) assist in S-protein mediated entry. This figure is taken from Wikipedia Commons under the Creative Commons Attribution-Share Alike 4.0 International license.

At the innermost layer of SARS-CoV-2 lies the nucleocapsid (N) protein which houses the viral genome. As previously mentioned, SARS-CoV-2 is a positive-sense RNA virus which allows it to translate its genome using host ribosomes immediately after entering the cell. The genome encodes 29 unique proteins. Encasing the nucleocapsid is the envelope (E) protein [15]. The membrane (M) proteins and the E protein form the complete viral envelope (Figure 1) [15]. On the surface of the envelope is the characteristic spike (S) protein, which the virus uses during attachment and entry into host cells [15]. The receptor for the S protein is angiotensin converting enzyme 2 (ACE2), which is expressed on a variety of cell types: alveolar cells, esophageal cells, absorptive enterocytes, myocardial cells, kidney proximal tubule cells, and others [16]. The S protein is primed by the cellular serine protease TMPRSS2, and the TMPRSS2 inhibitor camostat mesylate has been shown to impede SARS-CoV-2 infections in lung cells [17]. In TMPRSS2-negative cells, the cysteine proteases cathepsin B/L can facilitate S protein cleavage [17]. The N, E, M, and S proteins are the four structural proteins encoded by SARS-CoV-2 [15]. An overview of structural and non-structural (Nsp) SARS-CoV-2 proteins is provided in Table 1.

**Figure 2:**
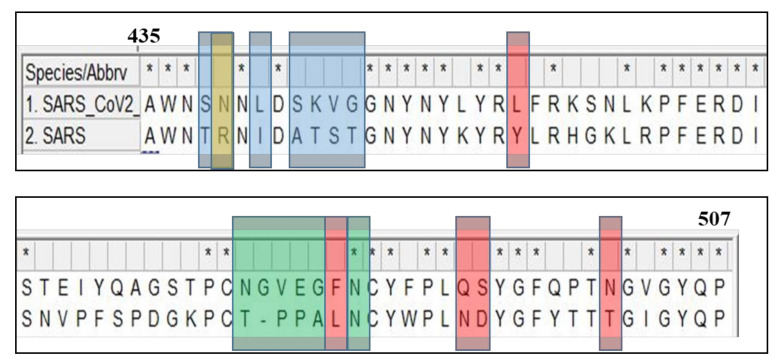
Schematic representation of amino acid sequence alignment of the receptor binding domain (RBD) of the spike glycoproteins of SARS-CoV and SARS-CoV-2. The highlighted regions are responsible for the structural change with following color codes: red, previously identified critical ACE2-binding residues; blue, the six RBM residues that differ between the SARS-CoV-2 wild-type RBD and SARS-CoV-2 chimeric RBD; yellow, a critical arginine on the side loop of the SARS-CoV RBM that forms a strong salt bridge with ACE2; and green, the residues on the variable loop between two disulfide-bond-forming cysteines in the ACE2-binding ridge.

The genome of SARS-CoV-2 shares approximately 80% of its sequence identity to SARS-CoV [18]. The 20% difference in sequences results in significant differences in structure and transmission of the virus. Sequence alignment comparisons for the S, M, RNA dependent RNA polymerase (RdRp), and helicase proteins for SARS-CoV-2, SARS-CoV, and MERS-CoV can be found in the Supplementary Figures (Figures S1–S8)). One of the most important variations in functionality occurs in the well-characterized spike protein. The SARS-CoV S protein is cleaved into two subunits during entry [19]. The S1 subunit contains a receptor binding domain (RBD) and attaches to ACE2 [19]. The S2 subunit then facilitates membrane fusion [19]. SARS-CoV-2 shares 76% identical amino acid identities for the entire S protein and only 74% for the RBD of its S1 subunit [20]. The amino acid mutations in the RBD from SARS-classic to SARS-CoV-2 include the following: Val^404^—Lys^417^, Tyr^442^—Leu^455^, and Leu^443^—Phe^456^ [21]. The RBD of the S protein has a more compact conformation in SARS-CoV-2 than SARS-CoV [22]. Moreover, several residue changes in the SARS-CoV-2 RBD stabilize two virus-binding regions at the RBD–ACE2 interface [22]. These structural features of the RBD of the S protein increase ACE2-binding affinity of SARS-CoV-2 (Figure 2) [22]. These changes are thought to cause a decrease in binding energy and a higher affinity for the SARS-CoV-2 spike protein to the ACE2 receptor [23]. The more favorable interactions may explain the increased transmission rate of SARS-CoV-2 compared to SARS-classic.

Interestingly, the C-terminal domain (CTD) of the SARS-CoV-2 S protein demonstrates a greater binding affinity for ACE2 than the RBD and contributes strong polar contacts to S protein–ACE2 interaction [24]. The CTD residue A475 interacts with ACE2 residue S19, N487 with Q24, E484 with K31, and Y453 with H34 [24]. Residue K417, located in helix α3 of the CTD core subdomain, was shown to contribute ionic interactions with hACE2 D30 [24]. Further virus–receptor contacts include SARS-CoV-2 CTD Y489 and F486 packing against ACE2 residues F28, L79, M82, and Y83 [24]. These contacts form a small patch of hydrophobic interactions at the interface.

3CLpro is a heterodimer composed of two chains A and B. Dimerization is a prerequisite for catalytic activity as each chain contains a N-finger domain, which interacts with glutamate residues on the sister chain [25]. These interactions provide structure to the catalytic site of the protease [25]. In contrast to the markedly distinct S proteins, the 3CLpro proteins of the two SARS viruses are quite similar. They share a 96% sequence identity, and the few differences largely reduce steric clash [26]. However, SARS-CoV-2 3CLpro has a slightly greater catalytic efficiency [25]. The 11 SARS-CoV-2 genomes found on the National Center for Biotechnology Information (NCBI) GenBank share nearly 100% conservation of 3CLpro sequences and the associated cleavage junctions on the replicase polyprotein [26]. Because of the conservation between SARS-CoV-2 strains and SARS-CoV, drugs that successfully target the 3Clpro of SARS-classic would be effective against the novel coronavirus. Unfortunately, no such drug candidates have entered Phase 1 clinical trials as of May 2020 [26]. Recently, certain α-ketoamide compounds demonstrated affinity to the 3CLpro protein and inhibited SARS-CoV-2 replication in a lung cell line [25].

The Nsps7–10 and Nsps12–14 of SARS-CoV-2 and SARS-CoV share over 95% structural similarity [15]. The most significantly different proteins (<80%) are Nsp3b and the spike protein [15]. Altogether, it appears that the group of proteins required for SARS-CoV-2 entry (S protein, ACE2, and TMPRSS2 and the Mpro) appear to be some of the most promising drug targets.

## 3. Viral Factors for Drug Development

### 3.1. Therapeutics Targeting Spike Protein

**Figure 3:**
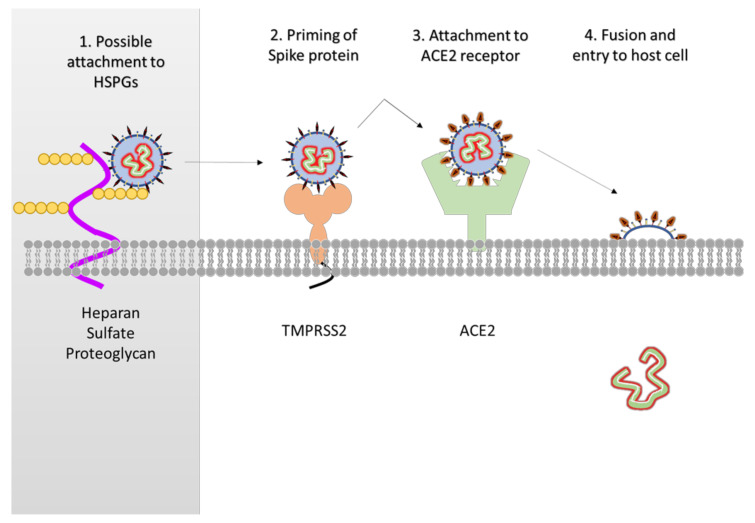
Overview of attachment and entry of SARS-CoV-2. While not confirmed in SARS-CoV-2, the original SARS-CoV required heparan sulfate proteoglycans (HSPGs) for attachment (1). The host enzyme TMPRSS2 primes and activates the S protein of SARS-CoV-2 (2). The virus uses the S protein to attach to the angiotensin converting enzyme 2 (ACE2) receptor (3) and initiate fusion and the release of its genome into the host cell (4).

Of the structural proteins, the S protein has been under the most scrutiny as a potential drug target given its essential role in viral entry and ease of access (Figure 3). Screenings performed by Wu et al. have identified multiple small-molecule compounds with high affinity to the S protein but only one, Hesperdin, was predicted to bind to the ACE2-binding region of the spike protein [15]. Xia et al. developed a lipopeptide called EK1C4, a derivative from the pan-coronavirus fusion inhibitor EK1, that inhibited the S protein-mediated membrane fusion of SARS-CoV-2 [27]. Either prophylactic or therapeutic treatment of EK1C4 conferred protection to mice from coronavirus infections, which is a powerful in vivo demonstration of the efficacy of EK1C4 as an antiviral compound.

### 3.2. Therapeutics Targeting NSPs

SARS-CoV-2 produces many Nsps as well. One of the most important ones is a 33.8 kDa protease known as the main protease (Mpro) or 3-chymotrypsin-like protease (3Clpro) [28]. Mpro cleaves and processes a replicase complex of two polyproteins pp1a and pp1ab at 11 sites to yield Nsp4–Nsp16 [28]. The papain-like protease (PLpro) cleaves the replicase polyprotein at its N-terminus to generate three Nsps, Nsp1–Nsp3 are essential for proper genome replication [29]. Both Mpro and PLpro are attractive targets for vaccine and drug development. Screening by Wu et al. indicated that a series of drugs such as Ribavarin, Valganciclovir, and β-thymidine bind to Mpro with high affinity [15]. Similar results have been found for the compounds Platycodin D, Chrysin, and Neohesperidin for PLpro [15]. Jin et al. discovered an irreversible inhibitor of Mpro called N3 [28]. N3 binds covalently to Mpro and impedes the functionality of its substrate-binding pocket [28]. Like NS3, the organoselenium compound Ebselen was also shown to inhibit SARS-CoV-2 infections in vitro [28]. Thus, researchers have reported multiple compounds of the Mpro and Clpro proteases with great potential as therapeutics.

### 3.3. RNA Dependent RNA Polymerase

SARS-CoV-2 Nsp12 is an RNA-dependent RNA polymerase which transcribes and replicates the viral genome [15]. The Nsp13 is a helicase which is essential for the replication of the coronavirus [15]. The inhibition of Nsp12 RdRp does not cause significant cellular toxicity to the host cells. However, there had been no specific inhibitors identified until recently [30]. Virtual screening has identified some potent drug molecules having anti Nsp12 effect. Some of the molecules such as anti-fungal drug Itraconazole, anti-bacterial drug novobiocin, chenodeoxycholic acid (drug for gallstones), anti-histaminic drug cortisone, anti-cancer drug pancuronium bromide, and anticoagulant drug dabigatran etexilate have been identified using computational software. Other than these chemical compounds, some naturally found products have binding affinity to RdRp such as betulonal from *C. xylocarpa*, gnidicin and gniditrin from *Gnidia lamprantha* [14]. In addition, vitamin B12 (methylcobalamin) may bind to the active site of the Nsp12 and potentially inhibit its function [31]. In another molecular docking study by Ruan et al., several presently available antiviral drugs were screened for their ability to inhibit the functions of RdRp. Saquinavir, Tipranavir, Lonafarnib, Tegobuvir, Olysio, Filibuvir, and Cepharanthine were selected on the basis of their docking score and binding free energies [32].

### 3.4. Molecules Targeting Helicase

The Nsp13 is a helicase which is essential for the replication of the coronavirus (15). Nsp13 can unravel double stranded DNA and RNA along the 5′ to 3′ direction in an ATP dependent process [33]. In fact, the Nsp13 of SARS-CoV is an important, conserved component for the replication of coronavirus. Hence, it has been selected as a target for antiviral drugs. There are a very few reports about Nsp13 inhibitor molecules [34,35]. According to an in vitro study by conducting DNA unwinding assay, Myricetin and Scutellarein are novel chemical molecules which may inhibit Nsp13 [36]. Another study on the Vero cell line merimepodib (MMPD) reported suppression of SARS-CoV-2 replication in vitro [37]. Based on modeling of the helicase protein structure, anti-bacterial drugs (lymecycline, cefsulodine and rolitetracycline), anti-fungal drug itraconazole, anti-human immunodeficiency virus-1 (HIV-1) drug saquinavir, anti-coagulant drug dabigatran, and diuretic drug canrenoic acid were predicted to be helicase inhibitors. Apart from these drugs many naturally occurring compounds like flavonoids and xanthose are predicted to have anti Nsp13 activity [15]. Finally, Nsp1, Nsp3c, and ORF7a, are viral proteins which play a role in inhibiting the host immune response [15]. Potential inhibitors for each of these proteins are described in this excellent study by Wu et al. [15]

### 3.5. Host Factors for Drug Development

Host factors may also be valuable therapeutic targets. Multiple coronaviruses including SARS-CoV-2 primarily utilize host heparan sulphate molecules to adhere to target cells prior to initiating attachment and entry [38,39,40,41]. This can be an attractive target given than multiple heparan sulphate inhibitors are currently available for testing [42,43] including a peptide molecule (G2) previously developed by our lab [44,45,46]. Additionally, heparanase, the only mammalian enzyme known to degrade polymeric heparan sulphate molecules, may play a significant role in viral entry and egress [47] and may be an attractive target against SARS-CoV-2. Furthermore, inhibition of the ACE2-S protein interaction impedes SARS-CoV-2 entry [48]. The authors introduced a human recombinant soluble ACE2 protein to infected Vero cells and demonstrated that it significantly reduced the recovery of the virus [48]. In addition, SARS-CoV-2 could infect engineered human blood vessel and kidney organoids, and this infection was abrogated by the soluble ACE2 protein [48]. The drug designed for the inhibition of host factors were originally developed for SARS-CoV. However, they can likely be applied to SARS-CoV-2 as the host factors have not changed. In a study by Adedeji et al., an HIV pseudotyped virus was designed with the surface glycoproteins of SARS-CoV and effects of various drugs acting on various host factors were studied. SSAA09E2 (N-((4-(4-methylpiperazin-1-yl) phenyl) methyl)-1,2-oxazole-5-carboxamide) acts through a novel mechanism of action, by blocking early interactions of SARS-S with the receptor for SARS-CoV, angiotensin converting enzyme 2 (ACE2). SSAA09E1 (((Z)-1-thiophen-2-ylethylideneamino) thiourea) acts later by blocking cathepsin L, a host protease required for processing of SARS-S during viral entry. SSAA09E3 (N-(9,10-dioxo-9,10-dihydroanthracen-2-yl) benzamide) also acts later and rather than affecting interactions of SARS-S with ACE2 or the enzymatic functions of cathepsin L, it prevents the fusion of the viral membrane with the host cellular membrane [49].

The TMPRSS2 is essential for entry and viral spread of SARS-CoV-2 due to its interaction with the ACE2 receptor. The TMPRSS2 inhibitor Camostat mesylate has been approved in Japan to treat unrelated diseases. It has been shown to block TMPRSS2 activity and thus can be taken as candidate molecule for treatment of COVID-19 [50]. Another approach for treatment is delivering excessive soluble form of ACE2 receptors. This will perform a dual function: slow viral entry into cells, thereby decreasing viral spread [51,52], and protect the lung from injury [52,53].

## 4. Therapies in Development

### 4.1. Remdesivir, Galidesivir, and Favipiravir

Ongoing research in drug therapies have yet to produce an approved drug. A comprehensive list of therapies in development is provided in Table 2. However, multiple drugs are undergoing trials. Gilead-backed remdesivir is currently undergoing two separate Phase 3 clinical trials in China for mild-to-moderate and severe COVID-19 (ClinicalTrials.gov: NCT04257656, NCT04252664) [54]. The former study was completed on April 10, 2020 while the latter has recently been completed. Remdesivir is a nucleoside analog with an unknown mechanism [54]. It is speculated to interfere with the activity of viral polymerases through termination of early RNA transcripts, with bolstered activity in mutant mice lacking the proofreading enzyme exoribonuclease [55]. Computer modeling of the SARS-CoV-2 RNA polymerase with remdesivir showed high affinity between the two molecules [56,57]. According to a nonrandomized compassionate-use study, a clinical improvement was observed in 36 of 53 patients (68%), the dose being 200 mg administered intravenously on day 1 and 100 mg daily for a remaining 9 days of treatment [58,59]. The Phase 3 study completed on April 10, 2020 in China did not show statistically significant results for time to clinical improvement [60]. However, as of May 2020, a National Institute of Allergy and Infectious Diseases (NIAID) report of the double-blind, controlled study shows a statistically significant decrease in recovery of patients after 4 days with remdesivir [61].

While remdesivir appears to be a frontrunner for drug development, several other drugs also show promise. In the same computer model, BioCryst drug galidesivir, an adenosine analogue, showed a similar affinity to remdesivir for binding to the SARS-CoV-2 RNA dependent RNA polymerase, matching the binding energy of native nucleotides [56]. The drug mechanism is similar to that of remdesivir in that it blocks the activity of viral RNA polymerase, prematurely terminating transcription [62]. In preclinical trials with SARS-CoV and MERS, galidesivir displayed antiviral activity and safety with healthy subjects [63]. The drug has begun Phase 1 trials for evaluation of safety and antiviral activity in subjects with yellow fever or COVID-19 (ClinicalTrials.gov: NCT03891420). The trial began on 9 April 2020 and is expected to conclude 31 May 2021.

Favipiravir (Avigan) is another nucleoside analogue that interferes with the action of RdRp [64,65]. While its mechanism of action is undetermined, it is thought to either induce deleterious mutations in RdRp, resulting in a nonviable phenotype, or selectively inhibit primer extension of RNA synthesis [66,67]. Favipiravir is currently undergoing a Phase 3 trial to evaluate safety and performance in patients with moderate COVID-19 (ClinicalTrials.gov: NCT04336904). The study began on March 25, 2020 and is expected to conclude in July 2020. Favipiravir is currently approved in Japan for treatment of the common influenza and in China for symptoms of COVID-19 [68,69]. Of note, coronavirus expresses an exonuclease enzyme (nsp14-ExoN) which has RNA proofreading capabilities which may mitigate the effects of nucleoside analogues. Previous studies have shown that nucleoside analog ribavirin has little effect on the coronavirus, leaving alternative therapies to be desired [69].

### 4.2. Experimental Therapy: Plasma Therapy

Plasma therapy, or plasmapheresis, has been used for over a century as a technique to filter blood extracorporeally in critical patients, eliminating cytokines and modulating their immune response. In recent years, convalescent sera have also been used to temporarily reduce the viral burden in H1N1 and Ebola patients as well as SARS and MERS [97,98,99]. One advantage plasma therapy may have over vaccinations is that, rather than relying on induction of immunity within the recipient, plasma therapy administers antibodies directly into the bloodstream [97]. The intended effect is virus neutralization although other mechanisms may be in effect, such as complement activation, antibody-dependent cellular toxicity, and/or phagocytosis [98]. The Food and Drug Administration (FDA) has already approved convalescent antibody therapy for COVID-19, and it has indicated potential prophylactic and therapeutic value [68]. A pilot study in China of 5 patients with severe COVID-19 reported clinical improvement from 4 days to 2 weeks post-transfusion although the study was constrained by small sample size and concomitant therapies [100]. While SARS-CoV-2 itself is not considered to be a transfusion-transmissible disease, the recipient takes a minor risk of contracting a bloodborne disease from the donor as well as transfusion-related acute lung injury (TRALI) and antibody-dependent enhancement of infection (ADE) [97].

### 4.3. Experimental Therapy: Hydroxychloroquine/Chloroquine

Six clinical trials are currently underway to evaluate the effectiveness of hydroxychloroquine (HCQ) in Mexico, South Korea, China, Spain, Norway, and the USA [59]. Hydroxychloroquine and analog chloroquine have been used historically to treat malaria as well as inflammatory conditions such as rheumatoid arthritis, lupus, and porphyria cutanea tarda [68]. Details of the drug mechanism of action are still being investigated. It is thought that the basic HCQ accumulates in the acidic lysosome and inhibits the degradation of external and internal cargo [101]. This mechanism would prove to be particularly useful in autoimmune disorders because it would prevent major histocompatibility complex (MHC) II autoantigen presentation. Chloroquine potentially inhibits pH-dependent stages of viral replication and interferes with an entry mechanism of SARS-CoV-2 [59]. The early study from China has shown some positive outcomes, albeit with small sample size (*n* = 30; ClinicalTrials.gov: NCT04261517). It is important to note that chloroquine and HCQ are associated with side effects such as vision loss, nausea, and possibly heart failure, so they are not recommended for prophylactic use [68]. These risks could be mitigated through use of an enantiomerically pure form of the drug as opposed to the typically administered racemic mixture of chloroquine stereoisomers although a full exploration remains desired [102]. A recent study found no benefit to treating patients with chloroquine or HCQ on in-hospital outcomes for COVID-19 [103].

### 4.4. Indomethacin

Previous studies found that indomethacin can inhibit the replication of several unrelated DNA and RNA viruses, including SARS-CoV. Cyclooxygenases (COXs) play a significant role in many different viral infections with respect to replication and pathogenesis [104]. Cyclopentone COX inhibitor indomethacin has been widely used in the clinic for its potent anti-inflammatory and analgesic properties [105]. Anti-coronavirus efficacy in vivo was confirmed by evaluating the time of recovery in canine coronavirus (CCV) infected dogs treated orally with 1 mg/kg body weight indomethacin [106].

### 4.5. Ivermectin

Ivermectin is an FDA-approved broad spectrum anti-parasitic agent [107]. In recent years, its antiviral properties of Ivermectin against many viruses have been observed [107,108,109]. To test antiviral property of Ivermectin against SARS-CoV-2, Vero/hSLAM cells were infected with SARS-CoV-2 isolate followed by addition of Ivermectin. The cells were harvested and tested by qRT-PCR. The viral load was reduced 93% after treatment with Ivermectin. After 48 h, the viral load was reduced by 99.98% with no significant cellular toxicity in the controls [107].

## 5. Vaccines in Development: mRNA-1273 and ChAdOx1 nCoV-19

Current observations indicate that coronaviruses are particularly adapted to evade immune detection and dampen human immune responses. This partly explains why they tend to have a longer incubation period of 2–11 days on average compared to influenza, 1–4 days. The longer incubation period is probably due to their immune evasion properties, efficiently escaping host immune detection at the early stage of infection. As a member of the Betacoronavirus genus, its immune evasion mechanism is potentially similar to those of SARS-CoV and MERS-CoV. The mechanisms by which SARS-CoV and MERS-CoV modulate host immune responses are described as follows. Aerosolized uptake of SARS-CoV-2 leads to infection of ACE2 expressing target cells such as alveolar type 2 cells or other unknown target cells. The virus may dampen anti-viral interferon (IFN) responses resulting in uncontrolled viral replication. The influx of neutrophils and monocytes/macrophages results in the hyperproduction of pro-inflammatory cytokines. The immunopathology of lung may be the result of the “cytokine storms”. Specific Th1/Th17 cells may be activated and contribute to exacerbate inflammatory responses. B-cells/plasma cells produce SARS-CoV-2 specific antibodies that may help neutralize viruses.

Coronaviruses interfere with multiple steps during initial innate immune response, including RNA sensing, signaling pathway of type I IFN production, signal transducer and activator of transcription (STAT) 1/2 activation downstream of IFN/IFN-alpha/beta-receptor (IFNAR) as indicated by suppressive marks. This delayed or dampening type I IFN responses impinge adaptive immune activation. Prolonged viral persistence exacerbates inflammatory responses that may lead to immune exhaustion and immune suppression as a feedback regulatory mechanism. Biased Th2 type response also favors poor outcome of the disease [110].

At present, there are no approved vaccines for SARS-CoV-2. However, the development process is being streamlined, and several vaccines have entered clinical trials. A list of vaccines in development is provided in Table 3. A nucleic acid vaccine, mRNA-1273, is currently in Phase 1 and expected to complete in June 2021 (ClinicalTrials.gov: NCT0428346) [54]. This project is sponsored by the US National Institutes of Health (NIH) through its Vaccine Research Center (VRC) in collaboration with Moderna. As of May 2020, Moderna has announced a successful Phase 1 testing of their mRNA-1273 and is expecting to begin Phase 3 testing in July [111]. mRNA-1273 encodes for a prefusion stabilized form of the coronavirus spike protein, a viral entry protein responsible for binding to host cell surface receptor ACE2 [57,59,112]. The vaccine is expected to introduce the mRNA code into host cells which will then begin producing S protein. The host immune system should recognize the product as foreign and subsequently develop a useful immune response in the event of an exposure. This method of inoculation has demonstrated a stronger CD8^+^ T-cell response than protein immunization and a more potent generation of antibodies in animals with lower doses than DNA immunization [113]. Despite these exciting prospects, it is important to note that there are no currently marketed mRNA vaccines, and mRNA-1273′s long-term effectiveness with humans is yet to be determined [112].

The Jenner Institute of Oxford University, in conjunction with pharmaceutical Advent Srl, is currently in Phase 1 and Phase 2 trials of evaluating an adenovirus vector vaccine ChAdOx1 nCoV-19 (ClinicalTrials.gov: NCT04324606). The study has a 6-month time frame with results to be completed in May 2021. ChAdOx1 nCoV-19 contains an attenuated form of the virus that produces S protein [68]. Upon exposure to the protein, the host should form antibodies which would provide immunity from SARS-CoV-2. As of May 2020, a preprint has emerged from the NIH and the University of Oxford which potentially demonstrates ChAdOx1 as effective in protecting six rhesus macaques from viral pneumonia [135]. The Jenner team behind ChAdOx1 nCoV-19 vaccine had previously worked on a vaccine for Middle East Respiratory Syndrome (MERS-CoV) from the same β-CoV genus [66]. Previous research with ChAdOx1 MERS in BALB/c mice models displayed high levels immunogenicity with a robust CD8^+^ T-cell response and high levels of neutralizing antibodies against the spike protein [136,137]. Subsequent Phase 1 trials reported ChAdOx1 MERS was safe and effective at inducing humoral immune responses in humans [138]. Given the high genetic similarity between MERS-CoV and SARS-CoV-2, some of the research conducted before the COVID-19 outbreak on MERS-CoV should translate to the novel coronavirus [139].

## 6. Conclusions

Given the current lack of treatments for SARS-CoV-2, there is a great demand to produce and scale therapeutics and vaccines to combat COVID-19. Before the current outbreak, there was not even a standardized treatment for the original SARS-CoV infections. Research and development are critically needed to protect against SARS-CoV-2 and future coronaviruses infections. In this review, we have discussed the epidemiology and structure of the novel coronavirus. We have also discussed two promising vaccine and three therapeutic treatments in development along with two experimental therapies that should be further investigated. If these treatments can be successfully developed and scaled, the length and severity of the COVID-19 outbreak could potentially be attenuated. Until then, social distancing and maintaining effective sanitization remain good tools for the public.

## Figures and Tables

**Table 1:** Overview of the name and functions of SARS-CoV-2 proteins. The four structural proteins and five of the most important Nsp are provided.

Sr. No.	Name of Viral Protein	Function
Structural Proteins		
1	Nucleocapsid (N) protein	Houses the viral genome.
2	Envelope (E) protein	Encases nucleocapsid and makes complete viral envelope along with M protein.
3	Membrane (M) glycoprotein	M and E protein makes complete viral envelope.
4	Spike (S) glycoprotein	On the surface of envelope, characteristic spike proteins. Attachment of virus with receptor at receptor binding domain (RBD). Cellular serine protease (TMPRSS2/Cathepsin B) cleaves S protein this facilitates viral entry.
**Non-structural proteins (Nsp)**		
5	Mpro	Also called as 3-chymotrypsin-like protease (3Clpro) It cleaves viral polyproteins pp1a and pp1ab at 11 sites to yield Nsp4 to Nsp16.
6	PLpro	It cleaves viral polyprotein at N terminus to yield Nsp1 to Nsp3
7	Nsp12	RNA dependent RNA polymerase Replication of virus
8	Nsp13	Helicase activity facilitates RNA dependent RNA polymerase (RdRp) during viral RNA replication
9	Nsp1, Nsp3c, and open reading frame (ORF) 7a	Inhibiting host immune response

**Table 2:** A comprehensive list of therapies in development for COVID-19.

Drugs in Trial		
Name:	Company:	Reference:
leronlimab	CytoDyn	[70]
Brilacidin	Innovation Pharmaceuticals	[71]
Gimsilumab	Roivant Sciences	[72]
TJM2	I-Mab Biopharma	[73]
AT-100	Airway Therapeutics	[74]
TZLS-501	Tiziana Life Sciences	[75]
OYA1	OyaGen	[76]
BPI-002	BeyondSpring	[77]
NP-120 (Ifenprodil)	Algernon Pharmaceuticals	[78]
APN01	University of British Columbia and APEIRON Biologics	[79]
Remdesivir (GS-5734)	Gilead Sciences	(ClinicalTrials.gov: NCT04257656, NCT04252664).
Actemra	Roche	[80]
Galidesivir	Biocryst Pharma	(ClinicalTrials.gov: NCT03891420)
REGN3048-3051 and Kevzara	Regeneron	[81]
SNG001	Synairgen Research	[82]
AmnioBoost	Lattice Biologics	[83]
Chloroquine	US FDA	[84]
Favilavir	Toyama Chemical	(ClinicalTrials.gov: NCT04336904)
Experimental Drugs		
Name:	Company:	Reference:
Existing antiviral compound library	Enanta Pharmaceuticals	[85]
Plasma-derived product candidates	Emergent BioSolutions	[86]
Ligand Epitope Antigen Presentation System (LEAPS) peptide immunotherapy	CEL-SCI	[87]
Hyperimmune globulin (H-IG) therapy	Takeda Pharmaceutical Company	[88]
Novel compounds for Therapy	Pfizer	[89]
Artificial intelligence (AI) platforms for drug discovery	Mateon Therapeutics	[90]
Rhodium virtual screening	Southwest Research Institute	[91]
Nanoviricide^®^ technology	NanoViricides	[92]
Monoclonal antibodies	Vir Biotechnology	[93]
HIV drugs for coronavirus treatment	Abbvie	[94]
LOPIMUNE, HIV Drug	Cipla	[95]
PREZCOBIX^®^ HIV medication (darunavir/cobicistat)	Janssen Pharmaceutical Companies	[96]

The names, associated company, and reference are given for each therapy. Both drugs in trial and experimental drugs are provided.

**Table 3:** A list of vaccines in development for COVID-19.

Vaccines in Development			
Name:	Company:	Platform	Reference:
Fusogenix DNA vaccine	Entos Pharmaceuticals	DNA vaccine	[114]
ChAdOx1 nCoV-19	University of Oxford	Adenovirus Vector	(ClinicalTrials.gov: NCT04324606)
AdCOVID	Altimmune	Intranasal Nasovax	[115]
Coronavirus vaccine	Medicago	Virus Like Particles	[116]
INO-4800	Inovio Pharmaceuticals and Beijing Advaccine Biotechnology	DNA vaccine	[117]
mRNA-1273 vaccine	Moderna and Vaccine Research Center	mRNA vaccine	(ClinicalTrials.gov: NCT0428346).
Avian Coronavirus Infectious Bronchitis Virus (IBV) vaccine	MIGAL Research Institute	Protein expression vector	[118]
TNX-1800	Tonix Pharmaceuticals	live modified horsepox vaccine	[119]
Recombinant subunit vaccine	Clover Biopharmaceuticals	Protein based Trimer vaccine	[120]
Coronavirus vaccine	Vaxart Inc	Oral recombinant Protein vaccine	[121]
Linear DNA Vaccine	Applied DNA Sciences and Takis Biotech	DNA vaccine	[122]
NVX-CoV2373	Novavax	Protein based	[123]
INO-4700	Inovio Pharma	DNA vaccine	[124]
Other Vaccines			
Name:	Company:		Reference:
AI Platform for the discovery and development of vaccines	Predictive Oncology	N/A	[125]
Shotgun Mutagenesis Epitope Mapping Platform	Integral Molecular	N/A	[126]
Develop antigens that mimic the native structures of the virus	AJ Vaccines	N/A	[127]
gp96 vaccine platform	Heat Biologics	N/A	[128]
B-cell and T-cell epitopes for Vaccine development	Hong Kong University of Science and Technology	N/A	[129]
COVID-19 vaccine	Vaccine by Generex	N/A	[130]
potential coronavirus vaccine	Vaccine by Tulane University	N/A	[131]
Cell Select™ and DeepDisplay™ discovery platforms	Coronavirus vaccine by ImmunoPrecise Antibodies	N/A	[132]
COVID-19 vaccine	Serum Institute of India	N/A	[133]
DNA vaccine/ recombinant measles virus vector -based vaccine	Zydus Cadila	N/A	[134]

The names, associated company, and reference are given for each vaccine. Both vaccines currently in development and other vaccines are provided. There are other ongoing trials that are not mentioned in this list.

## Supplementary Materials

The following are available online at https://www.mdpi.com/2077-0383/9/6/1885/s1, Figure S. Sequence alignment of the S protein of SARS-CoV-2 and SARS-CoV, Figure S2. Sequence alignment of the S protein of SARS-CoV-2 and MERS-CoV., Figure S3. Sequence alignment of the M protein of SARS-CoV-2 and SARS-CoV, Figure S4. Sequence alignment of the M protein of SARS-CoV-2 and MERS-CoV, Figure S5.: Sequence alignment of the RdRp protein of SARS-CoV-2 and SARS-CoV, Figure S6. Sequence alignment of the RdRp protein of SARS-CoV-2 and MERS-CoV, Figure S7. Sequence alignment of the helicase protein of SARS-CoV-2 and SARS-CoV, Figure S8. Sequence alignment of the helicase protein of SARS-CoV-2 and MERS-CoV
