# Supplementary material for: Vaccines and Therapies in Development for SARS-CoV-2 Infections
Source: J Clin Med. 2020 Jun 16;9(6):1885. doi: 10.3390/jcm9061885 (PMC7355822; doi:10.3390/jcm9061885)
Supplement: Supplementary file 1 [file jcm-09-01885-s001.pdf]

Supplementary Materials: The following figures contain comparisons of sequence alignments for SARS-CoV-2 (NCBI Reference Sequence: NC_045512.2), SARS-CoV (NCBI Reference Sequence: NC_004718.3), and MERS-CoV (NCBI Reference Sequence: NC_019843.3). The software MEGA-X was used to analyze sequence data. The color coding represents homology of the amino acids between the top and bottom sequence. For amino acid sequences the color coding is as shown below:

Symbol	Color	Symbol	Color
A	Yellow	M	Yellow
C	Olive	N	Green
D	Aqua	P	Blue
E	Aqua	Q	Green
F	Yellow	R	Red
G	Fuchsia	S	Green
H	Teal	T	Green
I	Yellow	V	Yellow
K	Red	W	Green
L	Yellow	Y	Lime

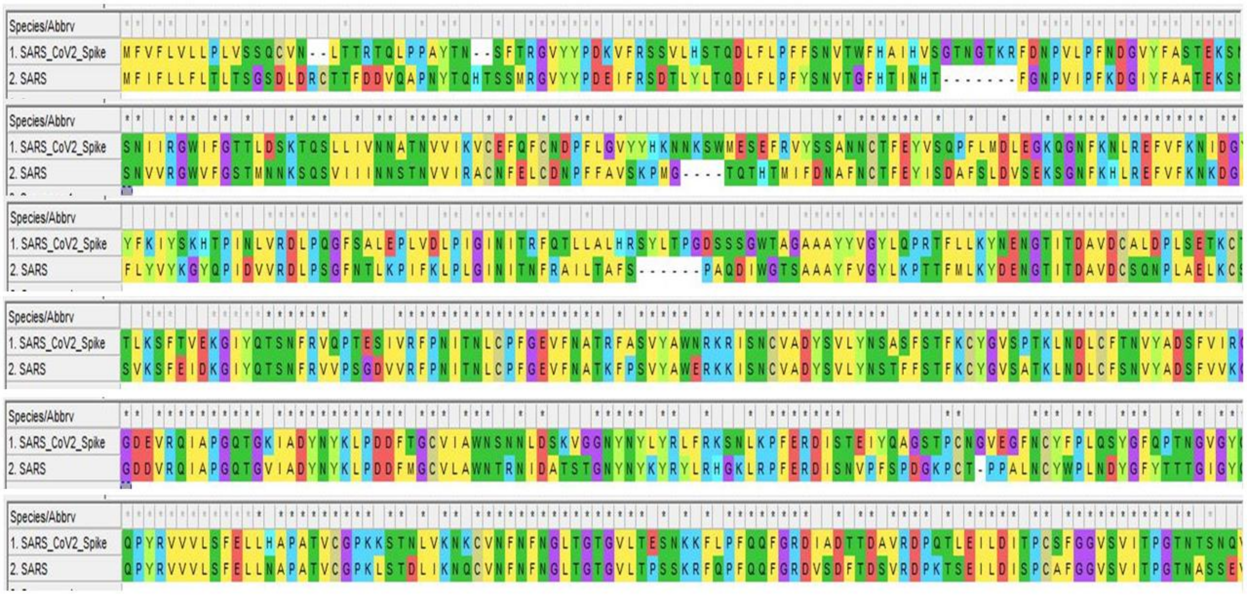

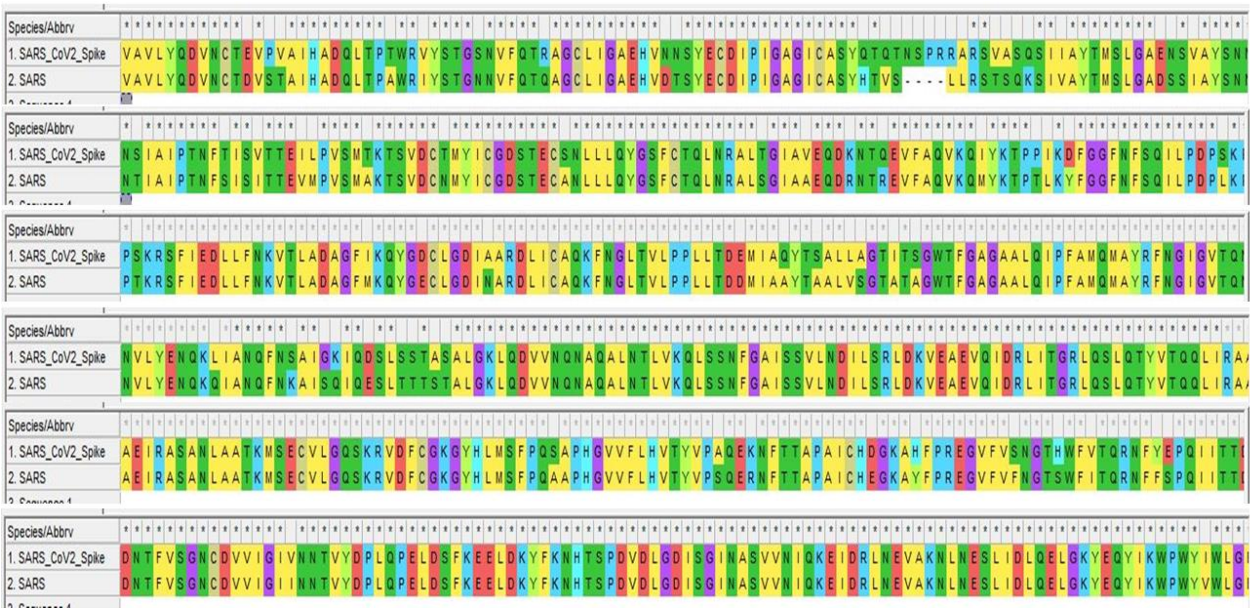

Figure S1. Sequence alignment of the S protein of SARS-CoV-2 and SARS-CoV.

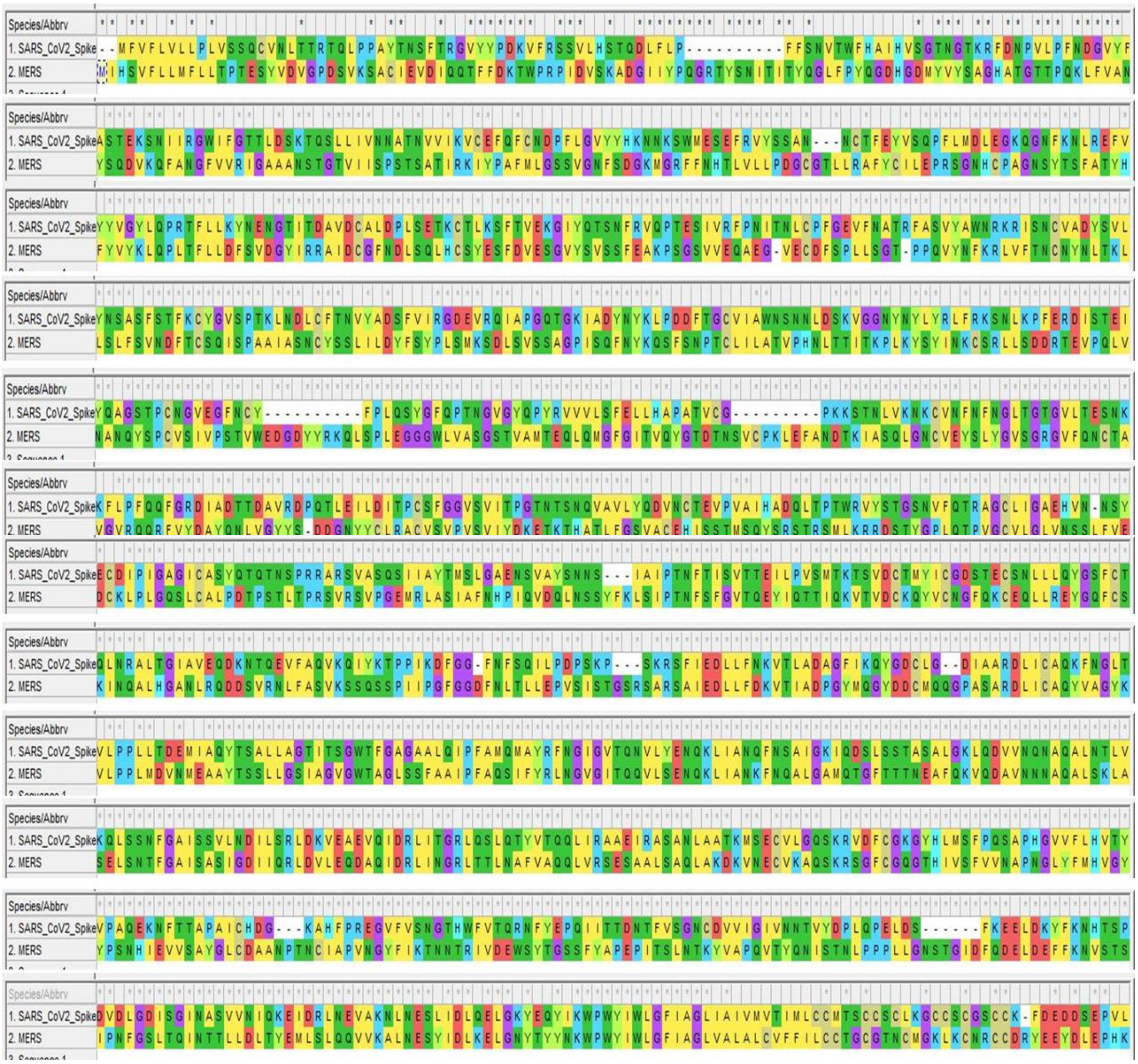

Figure S2. Sequence alignment of the S protein of SARS-CoV-2 and MERS-CoV.

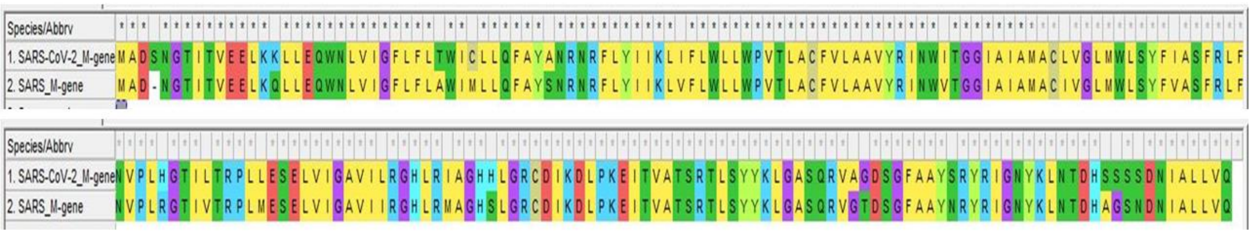

Figure S3. Sequence alignment of the M protein of SARS-CoV-2 and SARS-CoV.

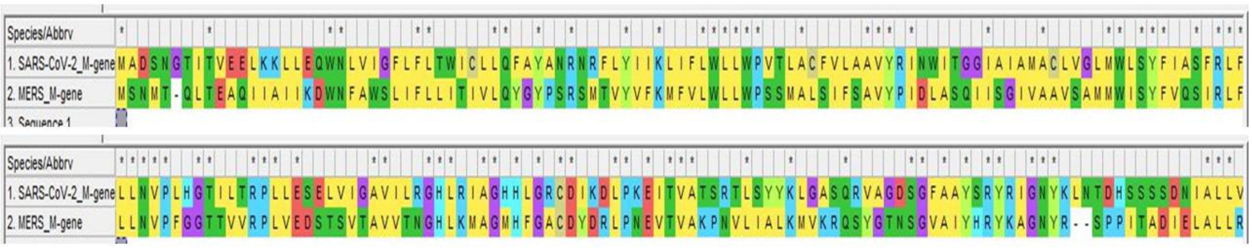

Figure S4. Sequence alignment of the M protein of SARS-CoV-2 and MERS-CoV.

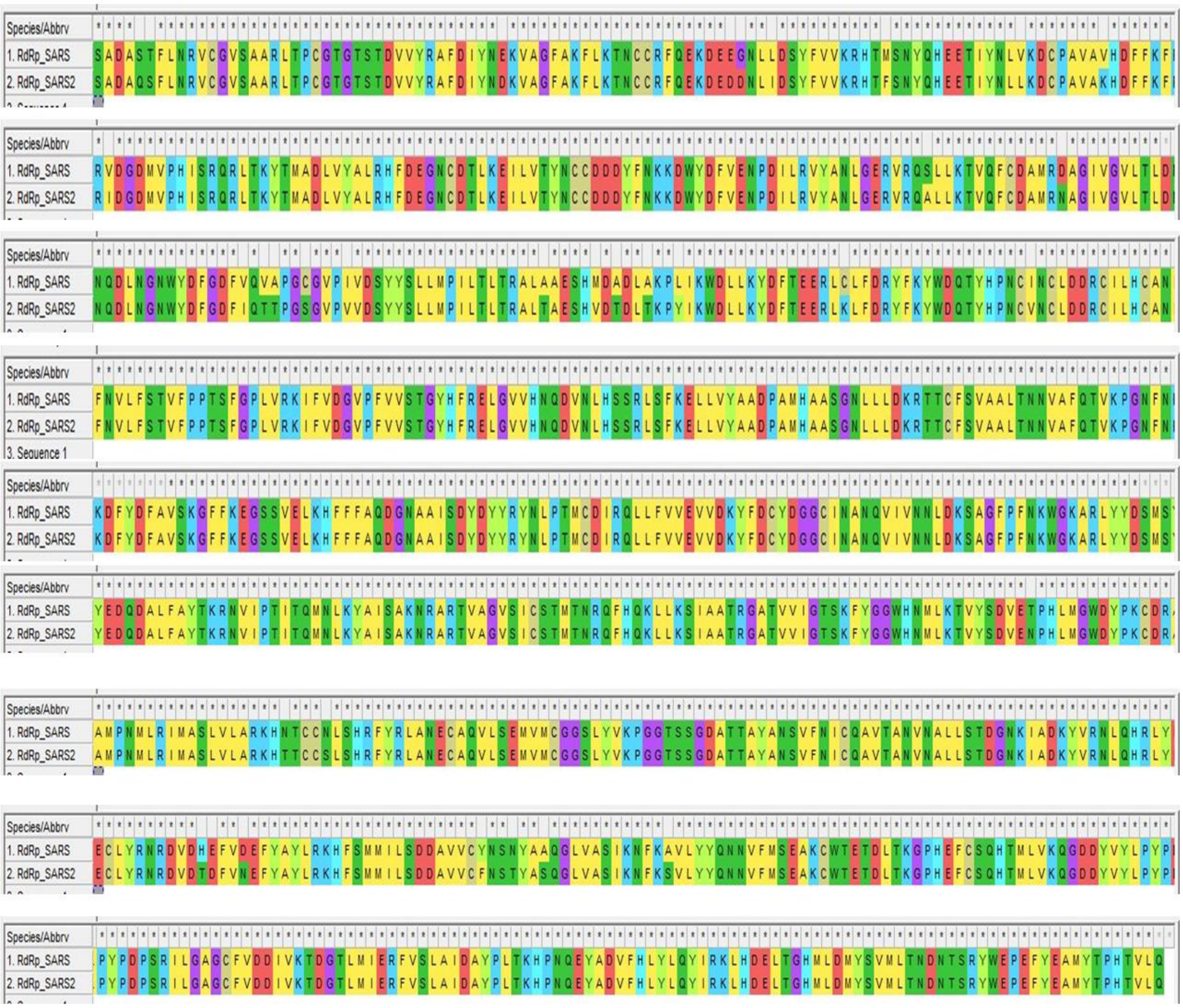

Figure S5.: Sequence alignment of the RdRp protein of SARS-CoV-2 and SARS-CoV.

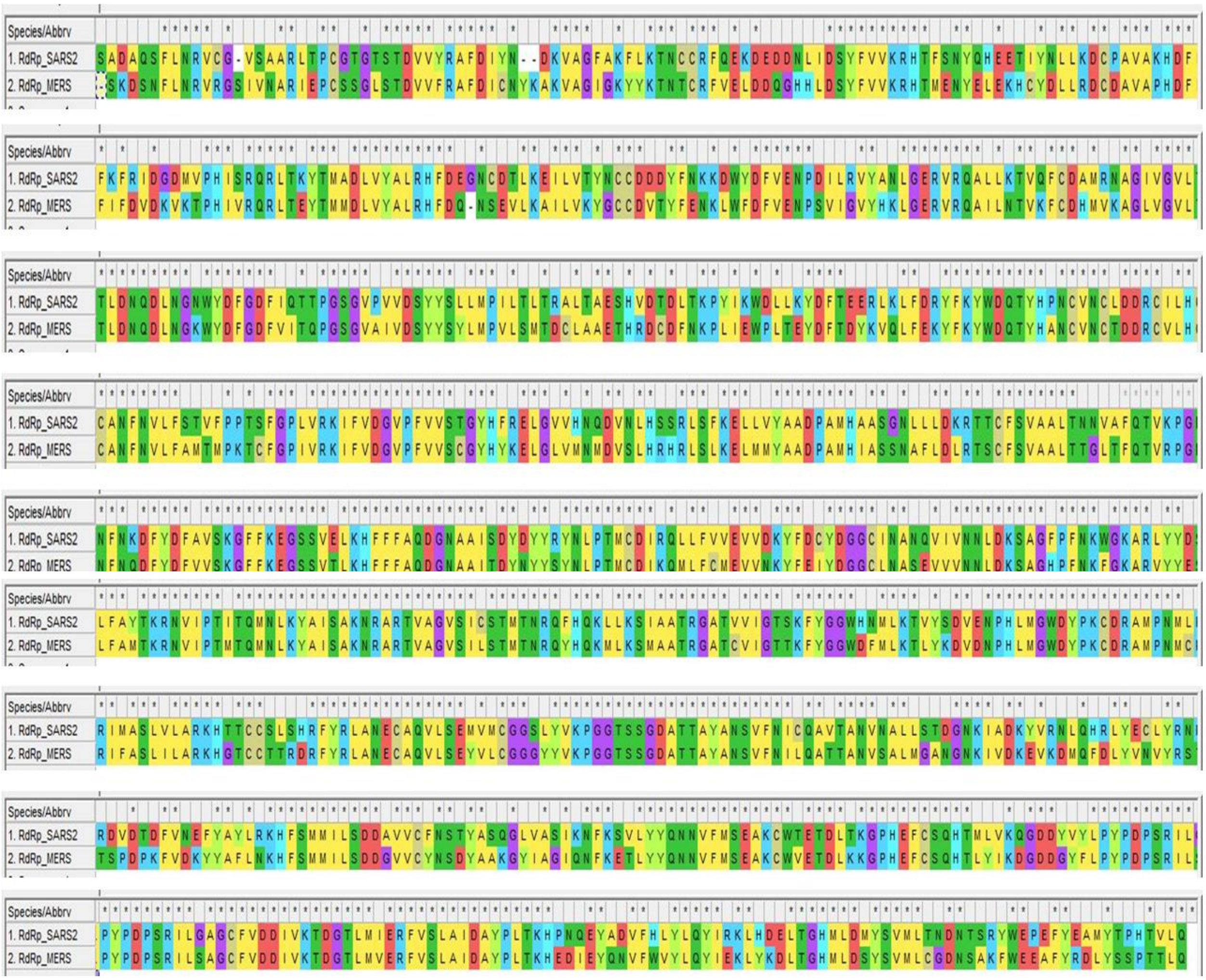

Figure S6. Sequence alignment of the RdRp protein of SARS-CoV-2 and MERS-CoV.

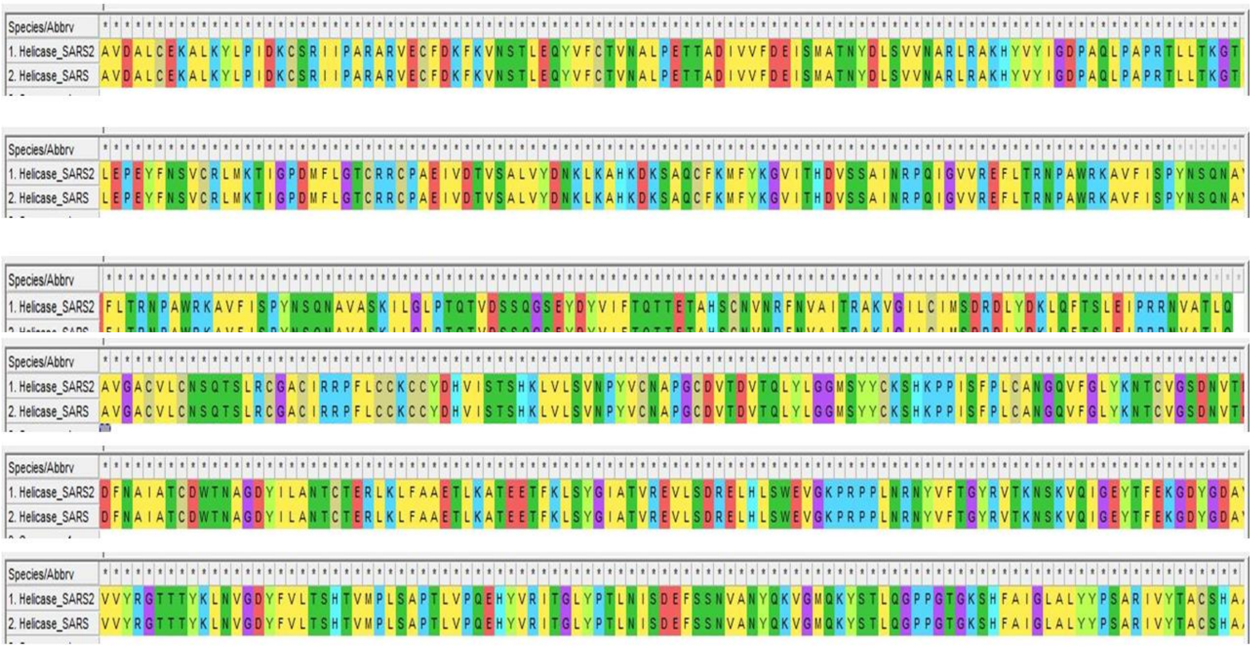

Figure S7. Sequence alignment of the helicase protein of SARS-CoV-2 and SARS-CoV.

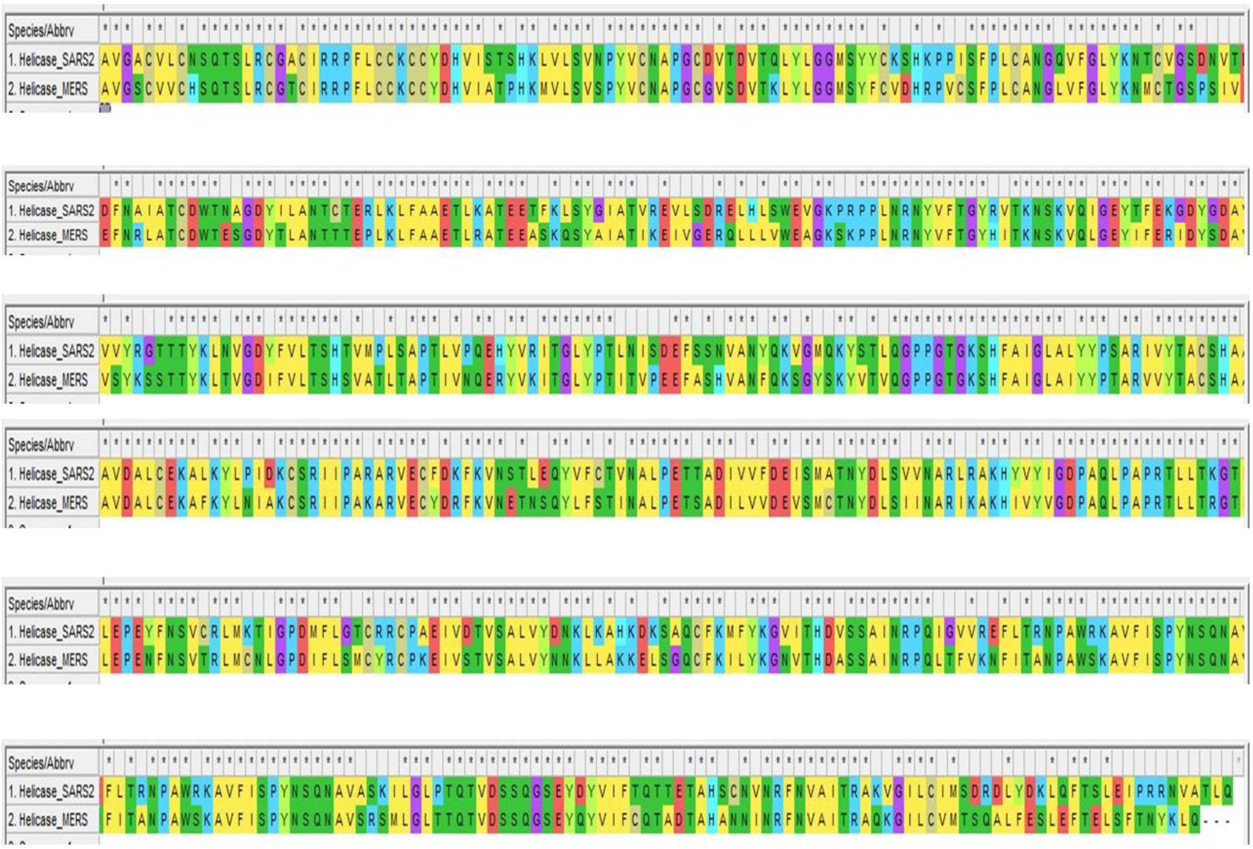

Figure S8. Sequence alignment of the helicase protein of SARS-CoV-2 and MERS-CoV.
